# Molecular Dynamics Simulation Study on the Influence of the Abrasive Flow Process on the Cutting of Iron-Carbon Alloys (α-Fe)

**DOI:** 10.3390/mi14030703

**Published:** 2023-03-22

**Authors:** Junye Li, Zhenguo Zhao, Junwei Li, Fujun Xiao, Rongxian Qiu, Hongcai Xie, Wenqing Meng

**Affiliations:** 1 Ministry of Education Key Laboratory for Cross-Scale Micro and Nano Manufacturing, Changchun University of Science and Technology, Changchun 130022, China; 2 School of Mechanical Engineering, Nanjing University of Science and Technology, Nanjing 210094, China

**Keywords:** molecular dynamics, nano-cutting, fluid medium, cutting angle

## Abstract

The plastic deformation behavior and microstructural changes in workpieces during ultra-precision machining have piqued the interest of many researchers. In this study, a molecular dynamics simulation of nano-cutting iron-carbon alloy (α-Fe) is established to investigate the effects of the fluid medium and cutting angle on workpiece temperature, friction coefficient, workpiece surface morphology, and dislocation evolution by constructing a molecular model of $C_{12}H_{26}$ as a fluid medium in the liquid phase using an innovative combined atomic approach. It is demonstrated that the presence of the fluid phase reduces the machining temperature and the friction coefficient. The cutting angle has a significant impact on the formation of the workpiece’s surface profile and the manner in which the workpiece’s atoms are displaced. When the cutting angle is 0°, 5°, or 10°, the workpiece’s surface morphology flows to both sides in a 45° direction, and the height of atomic accumulation on the workpiece surface gradually decreases while the area of displacement changes increases. The depth of cut increases as the cutting angle increases, causing greater material damage, and the presence of a fluid medium reduces this behavior. A dislocation reaction network is formed by the presence of more single and double-branched structures within the workpiece during the cutting process. The presence of a fluid medium during large-angle cutting reduces the number of dislocations and the total dislocation length. The total length of dislocations inside the workpiece is shorter for small angles of cutting, but the effect of the fluid medium is not very pronounced. Therefore, small cutting angles and the presence of fluid media reduce the formation of defective structures within the workpiece and ensure the machining quality.

## 1. Introduction

Steel is one of the most commonly used materials in industry and has become an essential component in people’s daily lives. The mechanical properties and applications of steel are determined by the Fe-C system, which is the foundation of steel. With the continuous development of industries such as aerospace technology, transportation, medical devices, automotive manufacturing and electrical engineering, manufactured steel parts are increasingly used in a wide range of applications. In particular, iron-based materials such as α-Fe are important for applications requiring high strength and good ductility structures in a variety of industries. Researchers are constantly investigating the precision and ultra-precision machining of materials in order to improve the performance of workpieces. The workpiece being machined will be accurate to the micron or even nanometer level during this process [1,2]. In practice, material removal may be limited to the workpiece’s surface, only a few layers of atoms or several atoms. However, such material processing or removal phenomena are difficult to describe in the macroscopic manner. Therefore, it seems necessary to study the behavior and mechanism of material deformation during the cutting process from the nanoscale.

Molecular dynamics (MD) is a popular atomic-scale research tool. Many scholars have recognized the scientific validity of MD because the generation and movement of dislocations during cutting processes can be studied using atomic dynamics simulations. In recent years, the use of molecular dynamics simulations to study the material processes occurring during machining has received great attention, and parameters such as machining surface [3], machining tool shape [4] and machining direction [5] have been widely discussed. Huan Liu et al. proposed an analytical model to predict the chip thickness and ploughing width of a FCC crystal in nanofabrication under an arbitrary crystal orientation, concluding that the crystal orientation determines the chip conversion between removal and ploughing, while the chip thickness and ploughing width can be predicted [6]. Yue et al. used the MD method to investigate a new nanostructured diamond abrasive for the mechanical polishing of single crystal silicon. The analysis showed that the structured abrasive leads to lower polishing forces and thinner subsurface damage layers in silicon polishing [7]. Ma et al. used MD simulations to investigated the crystal structure evolution and phase transition of single-crystal germanium materials during multiple cuts. A clear understanding of the brittle fracture, ductile plasticity and structural changes in single-crystal germanium materials was obtained at the atomic scale [8].

Many researchers are interested in the crystal orientation of BCC iron, using MD methods to simulate the effects of different crystal orientations on cutting, tensile behavior [9], impact phase transformation [10], nanoindentation and nano-scratching processes [11]. Li Xiang et al. found that, at the same defect concentration, different types of point defects cause different degrees of α-Fe lattice distortion and thus different ease of plastic deformation [12]. Wei wei et al. studied the effect of surface lithium atoms on the plastic deformation and yield stress of ferrite by suppressing the phase transition [13]. K Alhafez and Urbassek investigated the effect of different front angle tools on single crystal iron nanocutting using MD simulations [14]. Zamzamian et al. studied the mobility of 1/2 <1 1 1> {0 −1 1} edge dislocations in low carbon α-Fe [15]. Jiao et al. studied the effect of carbon on the deformation mechanism of iron–carbon alloys [16]. However, most of the research environments used in molecular dynamics to study the cutting characteristics of α-Fe are mostly in a vacuum, with a few exceptions in aqueous environments. This essay investigates the role of the liquid phase ($C_{12}H_{26}$) during abrasive flow cutting in a novel way, comparing it to liquid-free machining, and simulates the effect of the liquid phase on aspects such as abrasive grain motion and workpiece surface morphology changes at the microscopic atomic scale.

This study uses MD simulations to model SiC particles cutting iron–carbon alloy (α-Fe) workpieces in a liquid medium. The effects of the presence or absence of a fluid medium ($C_{12}H_{26}$) and cutting angle on the workpiece temperature, friction coefficient, workpiece surface morphology formation and workpiece atomic displacement mode during abrasive flow machining are investigated. This essay provides insight into the microscopic machining state and material removal phenomena in the presence of a fluid medium, which is of great academic significance and application value.

## 2. Materials and Methods

### 2.1. Model Building

**Figure 1:**
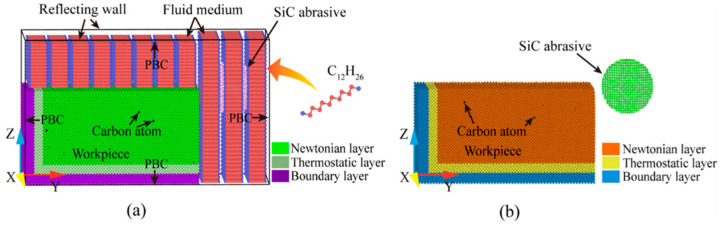
MD simulation system. (**a**) Wet cutting; and (**b**) dry cutting.

A molecular dynamics model of SiC particles cutting Fe-C alloys at room temperature was developed in this study to investigate the micro-cutting interaction between the abrasive particles and the workpiece at the atomic scale. In addition, two models were created, one with and one without a fluid medium, to investigate the effect of the fluid medium on the nano-cutting process. It is known that Fe maintains a body-centered cubic (BCC) structure at room temperature with a lattice constant of a = 2.867 Å. The overall dimensions of the simulation model are 11.452 nm × 17.178 nm × 8.589 nm (114.52 Å × 171.78 Å × 85.89 Å), with a total of 147,773 atoms, and the *x*, *y*, and *z* axes correspond to the crystal directions [100], [010], and [001]. We construct BCC Fe single crystal workpieces with a Newtonian layer, a thermostatic layer and a boundary layer for cutting, transfer, and stabilization. Furthermore, carbon atoms are randomly inserted into the Newtonian layer’s octahedral interstitial positions, allowing the carbon to reach its maximum solubility [17]. In this study, β-SiC abrasives with a sphalerite structure were used, which is widely preferred by technical processors due to its low price and polishing effect, comparable to that of diamond. SiC for cutting has a radius of 25 Å and a grain atomic number of 6287. To avoid initial interaction between the abrasive and the workpiece, the distance between the abrasive and the workpiece was set to 10 Å and the center of the abrasive was set flush with the workpiece surface. To simplify the model, the Moltemplate software was used to create a repetitive unit of $C_{12}H_{26}$ molecules as the fluid medium with a liquid phase molecular number of 62,064. The TraPPE-UA [18] force field was chosen to describe the intermolecular interactions of the fluid, and the joint atomic model was used to treat the ${CH}_{3}$ and ${CH}_{2}$ groups as a single interaction point to improve computational efficiency, while the hydrogen atoms in the ${CH}_{3}$ and ${CH}_{2}$ groups were only represented in the mass [19]. Figure 1 depicts the molecular dynamics model of Fe-C alloys cutting by SiC particles with and without a liquid medium at room temperature. The difference between the two models is only the relevant liquid medium setting. The specific model parameters are shown in Table 1.

### 2.2. Interatomic Potentials

In the simulation process, the potential function is related to the accuracy of the simulation, and the selection of the potential function is a very important part of the simulation process. The Modified Embedded Atom Method (MEAM) potential is an extension of the original EAM function, which can be well-applied to metals and alloys with BCC structures. In this study, the MEAM potential developed by Liyanage et al. is used to study the interaction between atoms in the FeC alloy workpiece, which can well-reproduce the gap energy of C atoms in the octahedral gap of BCC Fe [20]. In the MEAM potential function, the total energy E of the atomic system can be given by Equation (1):

(1) $$E=\sum_{i}\left\{F_{i}(\overset{-}{\rho_{i}})+\frac{1}{2}\sum_{i\neq j}\emptyset_{ij}\left(r_{ij}\right)\right\}$$

where F is the embedded energy as a function of the atomic electrical density ρ. ∅ is a pair of potential interactions, which is the sum of all neighbors j of atom i within the cut off distance r.

The Morse potential function can be effectively applied to the deformation of cubic metals [21]. Therefore, we use the Morse potential function to describe the interaction between the Fe-C alloy workpiece and the SiC abrasive. In the Morse potential function, the potential energy $\varphi_{ij}$ of two atoms i and j with a distance of $r_{ij}$ is given by Equation (2):

(2) $$\varphi \left(r_{ij}\right)=D\left[e^{-2\alpha \left(r_{ij}-r_{0}\right)}-{2e}^{-\alpha \left(r_{ij}-r_{0}\right)}\right]$$

where D is the binding energy coefficient, α is the gradient coefficient of the potential energy curve and $r_{0}$ is the equilibrium distance between the two atoms.

The Lennard–Jones (L-J) potential function is usually used for the simulation of the liquid environment [19]. In this study, the micro-cutting process in an organic medium is simulated, and the potential function is selected to describe the interaction involving organic molecules. The potential energy $U_{L-J}$ is given by Equation (3):

(3) $$U_{L-J}\left(r\right)=4ℇ\left[{\left(\frac{\sigma }{r}\right)}^{12}-{\left(\frac{\sigma }{r}\right)}^{6}\right]$$

where σ is the equilibrium distance between atoms when the interaction potential is 0. ℇ is the depth of the potential well, which reflects the strength of the mutual attraction between two atoms at a distance of r.

### 2.3. Simulation Environment

**Figure 2:**
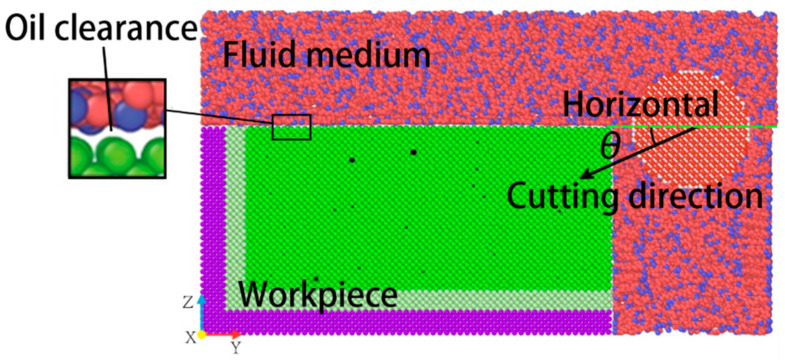
Longitudinal-sectional view of the system model after relaxation.

The MD simulation of the cutting process is divided into a chilling phase and a machining phase. In particular, the relaxation phase is used to bring the initial simulated system to a steady state. In this study, the energy minimization process was carried out using the conjugate gradient method, and the equilibrium constraint of 100 fs was applied to the system in a Nose–Hoover heat bath during the relaxation phase to keep the temperature at 293 K. After the relaxation, the system remains intact without any collapse. Figure 2 depicts the model section of the system after relaxation.

After relaxation, a stable oil clearance exists between the liquid phase molecules and the workpiece. The fluid molecules have strong self-diffusion and fill the whole free space above and on the right of the workpiece. As can be seen from the figure, the workpiece is wrapped in fluid molecules to form a dense oil film. In the simulated processing stage of abrasive flow, the abrasive grain is set as a rigid body. According to the previous research and calculation of abrasive flow processing, the motion of the abrasive grain is set to 50 m/s according to the actual processing speed, and the depth of cut is 1.25 nm. The cutting is performed along the *y*-axis with cutting angles of 0°, 5°, and 10° to the workpiece. The cutting distance is 11 nm, the cutting crystal direction is (001) [0–10], and the integral step is 1 fs. During machining, the temperature and energy in the system change continuously as the simulation proceeds, but the volume basically does not change. Therefore, the NVE system is used to balance the simulation system. In the process of solid–liquid two-phase abrasive flow, aviation kerosene or hydraulic oil are usually used as the fluid phase of the liquid phase. The fluid medium in this study is aviation kerosene. Aviation kerosene is a mixture of large molecule hydrocarbon complex hydrocarbons with a complex composition, and $C_{12}H_{26}$ is an important component of aviation kerosene, so it is used as a cooling fluid ($C_{12}H_{26}$ is a colorless liquid with a melting point −9.6 °C, boiling point 216.3 °C, flash point 71 °C, density 0.753 g/cm^3^, vapor pressure 0.133 kPa/47.8 °C, is insoluble in water, and has good heat dissipation). The Periodic Boundary Conditions (PBC) are set along the X and Y direction for the entire system. Fixed boundary conditions along the Z direction and the reflective wall technique are used to keep the density and pressure of the fluid phase constant. The fluid medium appears to play a larger role in wet and dry cutting with abrasive flow [22]. Therefore, this study explores the effect of the existence of the fluid phase on machining by comparing wet and dry cutting. The initial simulation conditions are similar to the environmental model without the liquid phase. The difference between the two simulations is in the liquid phase environment setting.

In this study, the Large-scale Atomic/Molecular Massively Parallel Simulator (LAMMPS) [23] code is used for MD simulation of SiC abrasive cutting Fe-C alloy. The dislocation extraction algorithm (DXA) [24] is used to identify lattice dislocations, which allows us to determine their Burgers vectors. We used the free software Open Visualization Tool (OVITO) [25] for visualization. In addition, the elastic constants of α-Fe using this EAM potential were $C_{22}=212.84\;GPa,\;C_{13}=143.23\;GPa,\;C_{66}=118.18\;GPa$. These values were very close to the elastic constants $C_{22}=219\;GPa,\;C_{13}=146\;GPa,\;C_{66}=123\;GPa$. Additionally, we calculated by density functional theory (DFT) for the iron–carbon alloy [26]. The above two tests show that the EAM potential function can correctly express the physical properties of α-Fe.

## 3. Results and Discussion

### 3.1. Analysis of Liquid Phase Flow State and Effect

#### 3.1.1. Analysis of Fluid Medium Flow State

**Figure 3:**
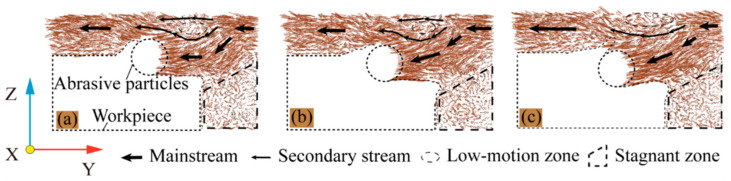
The instantaneous movement distributions of the fluid medium at a cutting distance of 80 Å. (**a**) θ = 0°; (**b**) θ = 5°; and (**c**) θ = 10°.

In the machining process, the fluid medium plays an important role. After relaxation, the workpiece, the abrasive particle and the fluid medium all remain in a stable state. When the abrasive particles move in the machining direction, the fluid molecules also flow around the abrasive particle and the workpiece. Given the size of the simulation cell and the simulated process time, the influence of the fluid on the process must be rated as high. Therefore, in order to study the movement of the fluid medium during the machining process, the instantaneous motion distribution of the fluid medium at different cutting angles at a machining distance of 80 Å was selected for this study. The transient motion of the fluid medium during cutting is shown in Figure 3.

As can be seen from Figure 3, the fluid medium movement in the abrasive flow is primarily driven by the abrasive particles. The movement in the path through which the abrasive particles pass is the dominant flow, the other mainstream is the flow of fluid molecules attached to the unprocessed surface of the workpiece. The fluid is attached around the workpiece and the abrasive particles. The fluid constantly exchanges heat with the workpiece at all times, which can remove some of the cutting heat generated by the machining. This flow can reduce the increase in workpiece temperature caused by partial processing and reduce the tool wear. The secondary flow and low motion zone, which is primarily above the abrasive particle, is caused by the fact that the movement of the abrasive particle is insufficient to drive the fluid there in a directional flow. Because of the workpiece obstruction and less interference, a stagnant flow zone forms on the right side of the workpiece. As a result, some of the fluid remains inside the workpiece during the machining process, causing losses. As the cutting angle increases, the flow of fluid molecules penetrates deeper into the workpiece with the abrasive grain. To achieve lubrication, the fluid medium surrounding the abrasive particle can reduce friction between the abrasive particle and the machined surface as well as friction between the abrasive particle and the chips. The increase of the cutting angle has a greater influence on the movement direction of the mainstream in the part that moves with the abrasive particle, but has less influence on the flow state of fluid molecules in other areas.

#### 3.1.2. Effect of Fluid Medium on Workpiece Temperature

**Figure 4:**
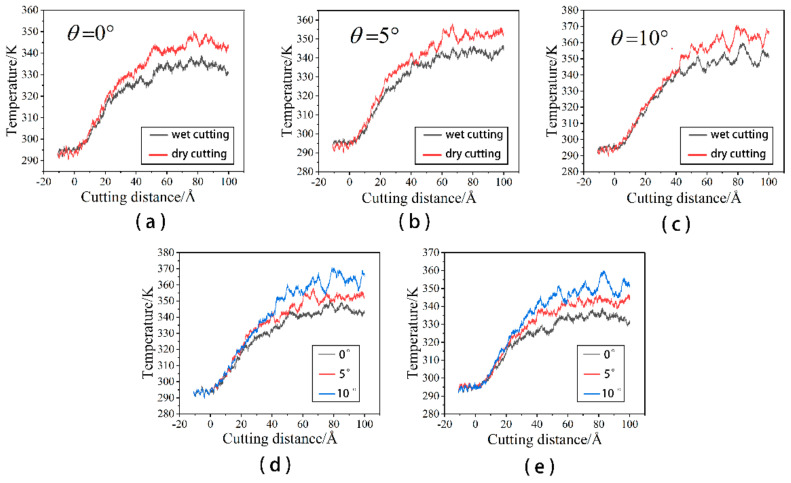
The temperature change of the workpiece. (**a**) θ = 0°; (**b**) θ = 5°; (**c**) θ = 10°; (**d**) workpiece temperature change during dry cutting; and (**e**) workpiece temperature change during wet cutting.

Cutting heat is produced during the machining process, which is one of the most important physical phenomena in machining. The majority of the energy consumed during machining is converted into heat, except for a very small proportion of the energy used to form new machined surfaces and new lattice deformations. Therefore, we could say that all of the energy consumed during machining is converted into heat. The large amount of cutting heat makes the workpiece temperature rise, which will directly affect the performance of the workpiece material, workpiece processing accuracy, and the quality of the machined surface. At the same time, The temperature also has an effect on the reaction between the dislocations and dislocation rings within the BCC Fe. Relative to dry cutting, the presence of a fluid medium reduces the internal temperature of the workpiece by dissipating heat. The temperature change in the Newtonian layer of the workpiece was recorded for both cases with and without fluid molecules to better characterize the cooling effect of the fluid phase on the workpiece. The workpiece temperature variation with and without fluid media at different cutting angles is shown in Figure 4.

When the cutting angle is 0°, the cutting distance at which the abrasive particle starts to act on the workpiece is set to 0 Å. As can be seen from Figure 4a–c, the presence of the fluid medium has a positive effect on the heat dissipation of the workpiece during the machining process at different chip angles. At the beginning of the machining process, the abrasive grains are only in contact with the workpiece. Because the number of chips produced and the number of lattice transformations are low, the workpiece temperature rises more slowly. At this point, the liquid phase’s cooling effect is not visible. On the contrary, due to the friction between the abrasive grain and the fluid medium, the temperature of the fluid medium increases and is transferred to the workpiece. Therefore, in the initial stage of cutting, the cutting process with the fluid media workpiece temperature is higher than the workpiece temperature during dry cutting. The temperature of Newtonian layer atoms rises rapidly as the cutting distance increases. On the one hand, this is partly due to the extrusion of the workpiece atoms by the abrasive particles and the disruption of the chemical bonds between the workpiece atoms. On the other hand, as the lattice strain energy stored is released, some of the released energy is converted into heat. All of this contributes to the workpiece’s temperature rapidly rising. The workpiece is severely damaged at this point, and the contact area between the liquid phase and the workpiece expands. The cutting heat in the workpiece and chips is transferred through a large number of liquid phase molecules, which slows down the temperature rise of the workpiece. As can be seen from Figure 4d,e, the temperature of the workpiece increases with increasing cutting angle during wet cutting and dry cutting. This is due to the fact that the large cutting angle increases the degree of workpiece destruction and more atoms release heat. The cut reaches stability in the later stages of cutting, and the number of atoms destroyed and lattice transitions is relatively constant, so the workpiece temperature gradually remains constant. When comparing the work-piece temperature during the stable cutting period, the presence of a fluid medium can reduce the work-piece temperature by approximately 10 K. According to previous research, the presence of cutting fluid in the mechanical cutting process can better reduce the cutting temperature of the conclusion [27,28]. This study is an important resource for explaining the reasons under a microscopic perspective.

#### 3.1.3. Effect of Fluid Medium on Friction Coefficient

During the cutting process, the abrasive particle will rub against the chips or the machined surface, thus impeding the movement of the abrasive particle. The presence of the liquid phase mitigates this effect. In order to characterize the lubrication effect of the presence of the liquid phase on the machining process, we calculate the change of the friction coefficient during the machining process. For the machining with a cutting angle of 0°, the friction coefficient is defined as the ratio between the tangential force and normal force [29]. For the angular cutting in this study, the friction coefficient will be given by Equation (4):


(4) $$\mu =\frac{F_{t}}{F_{n}}=\frac{F_{y}\cos\theta +F_{z}\sin\theta }{F_{z}\cos\theta -F_{y}\sin\theta }$$

**Figure 5:**
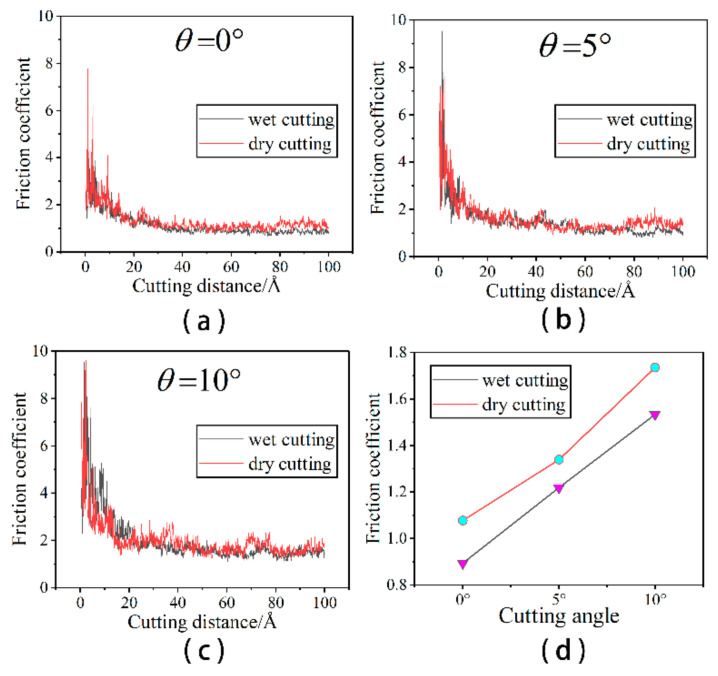
The change of friction coefficient. (**a**) θ = 0°; (**b**) θ = 5°; (**c**) θ = 10°; and (**d**) comparison of friction coefficient during wet and dry cutting at various cutting angles.

The variation of the friction coefficient at different cutting angles is shown in Figure 5.

**Figure 6:**
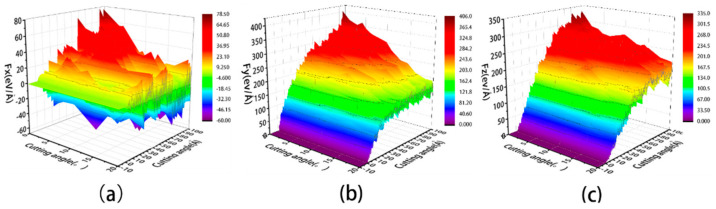
Variation of cutting force with cutting distance. (**a**) Fx; (**b**) Fy; and (**c**) Fz.

As can be seen from Figure 5a–c, the friction coefficient without a liquid phase is slightly higher than that with a liquid phase at the same cutting angle. In the early stages of cutting, when the abrasive grain first contacts the workpiece, a large tangential force is required to displace some of the workpiece atoms in the front of the abrasive particle and build up to produce the chips and measured flow. The resulting machined surface is small at this point, as is the normal force required to form the machined surface. As a result, tangential and normal forces increase in different ways during the preliminary cutting process. In addition, the coefficient of friction fluctuates considerably in the early stages of cutting due to the vibrations caused by the continuous collision between the abrasive grains and the workpiece. The larger friction coefficient ensures that the abrasive grains overcome the interaction forces between the atoms of the workpiece and begin to have a damage the workpiece. As the cut proceeds, the chip formation pattern enters a steady state. The variation of the tangential and normal forces smooths out, as does the value of the friction coefficient. In order to see more visually the effect of the presence of the fluid medium and the cutting angle on the machining, we calculated the mean values of the coefficient of friction after a 2.5 nm cutting distance, as shown in Figure 5d. Figure 5d shows that the presence of the fluid medium reduces the friction coefficient, which reduces the frictional resistance and energy loss. This is also consistent with previous research, which concluded that cutting forces and frictional wear are better reduced when a cutting fluid is present [30,31]. This essay provides a more detailed explanation of the causes of this. The coefficient of friction increases with the increasing cutting angle. Larger cutting angles cause more increased friction between the abrasive grain and the workpiece, resulting in higher cutting forces and friction coefficients. This study examines the changing state of cutting forces under wet cutting conditions at microscopic angles to further investigate the relationship between cutting forces and cutting angles. At the microscopic scale, the cutting force is the force between the particle atoms and the workpiece atoms. It is the cutting force of the Si and C atoms in silicon carbide particles on the Fe and C atoms in the workpiece. The cutting force between the particle atoms and the workpiece atoms indirectly reflects the micro-cutting action of the abrasive particles and the material removal process, and it is an important physical parameter in the micro-cutting process, as illustrated in Figure 6.

As can be seen in Figure 6a, the cutting forces on both sides of x cancel each other out, causing the Fx values to be distributed around 0. The lattice structure on both sides is not consistent after the abrasive grain has cut through the workpiece due to the random distribution of C atoms inside the workpiece, which also contributes to the deviation of Fx up and down at a certain cutting distance. As shown in Figure 6b, the Fy value increases faster at the beginning of the cutting process and gradually smooths out at a later stage. When the cutting angle is 0°, the cutting distance is approximately 50 Å. The abrasive grain has been completely cut into the workpiece at this point, and the cutting process has reached a stable cutting stage, where the Fy value has reached its peak and is stable. The Fy value gradually increases as the cutting angle increases, especially in the middle and later stages of cutting. When the cutting angle is 15° or 20°, Fy continues to rise in the late cutting stage, making it more difficult to achieve the stable cutting stage. If the cutting angle is too large, it is difficult to discharge the chips formed after the abrasive grains enter the workpiece, causing the cutting action to be hampered. As shown in Figure 5c, Fz increases approximately linearly in the early stages of cutting, slowly after the grain has been completely submerged in the work-piece, and then gradually stabilizes in the later stages. Fz allows the atoms beneath the grain to form a machined surface while also overcoming the chip’s obstruction to the grain. Fz increases as the cutting angle increases at the same cutting distance. Abrasive grains with a higher cutting angle create deeper cuts in the workpiece, increasing the number of atoms contacted by the grain. To force plastic deformation of the material in the cutting area, the particles must overcome greater bonding energy and break more atomic interactions. As a result, as the cutting angle increases, the abrasive grain cutting forces increase. This is also consistent with previous research, which concluded that increasing cutting angles increases cutting forces within a certain range [32]. Therefore, smaller cutting angles and the presence of fluid improve the quality of the machined workpiece. 

### 3.2. Evolution of Workpiece Surface Morphology

**Figure 7:**
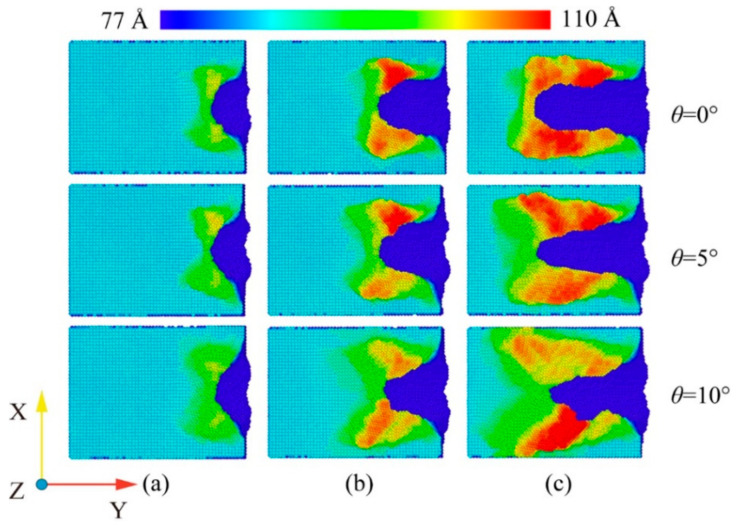
Workpiece surface morphology during wet cutting. (**a**) Cutting distance of 30 Å; (**b**) cutting distance of 60 Å; and (**c**) cutting distance of 100 Å.

**Figure 8:**
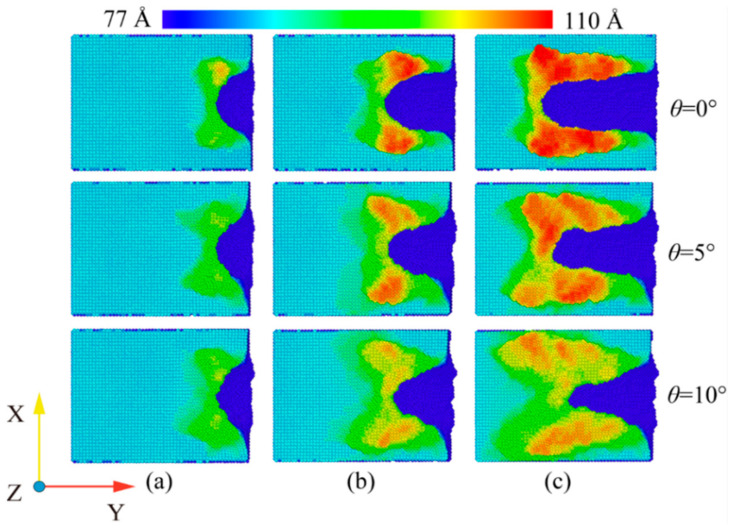
Workpiece surface morphology during dry cutting. (**a**) Cutting distance of 30 Å; (**b**) cutting distance of 60 Å; and (**c**) cutting distance of 100 Å.

At room temperature, the Fe maintains the BCC lattice structure. Even if a small amount of carbon atoms enter the octahedral gap positions, the workpiece still remains cubic. Under conditions free of external forces, the atoms inside the workpiece are arranged in an orderly fashion and the lattice structure remains intact. When the abrasive grains machine the workpiece, a build-up of workpiece atoms begins to form on the workpiece surface. At the same time, some areas of the workpiece surface are extruded and deformed, resulting in a substantially altered surface. In order to study the effect of fluid media and different cutting angles on the surface quality of Fe-C alloy workpieces, the surface morphology was observed for cutting processes with and without fluid media selected for cutting distances of 30 Å, 60 Å and 100 Å. The workpiece atoms were colored based on their height along the *z*-axis. At the microscopic size atomic model scale. The surface roughness rule is rarely used because surface roughness is difficult to observe at the microscopic level and data quantification accuracy is low. The roughness level of a workpiece surface is typically calculated by observing the height of the surface bulge as well as the number and arrangement of atoms. Figure 7 depicts the surface profile of the workpiece with fluid lubrication, where the fluid medium and abrasive particles are hidden. Figure 8 depicts the workpiece’s surface profile without fluid media.

**Figure 9:**
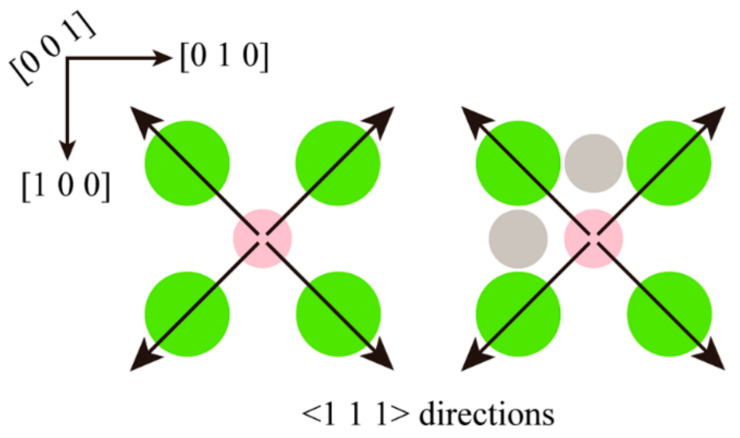
Schematic diagram of the crystal slip direction during cutting. The big green circles (small pink circles) represent the first (second) layer of atoms. The gray circles represent the carbon atoms partly in the octahedral gap. The arrows indicate the <1 1 1> slip direction.

The silicon carbide fragments that cut the iron–carbon alloy form an approximately symmetrical pile-up on the (0 0 1) surface. Figure 7 and Figure 8 show that the chips do not gather in front of the abrasive particle, but rather flow to both sides at 45°. The chip atoms are roughly symmetrically distributed on both sides of the groove formed by the abrasive particle’s passage. The slip characteristics of the BCC Fe structure determine this build-up behavior, which is unaffected by the presence of fluid media or changes in the cutting angle. Figure 9 shows the slip direction of the BCC Fe crystal. These pile-up features result from the fact that the <1 1 1> slip directions point at 45° to the sides for the cutting process. The number of carbon atoms is small and they are not in the sliding direction, which has little influence on the formation of the pile. However, there is a slight asymmetry in the pile-up due to the atomic and irregular nature of the dislocation generation and reaction process. At the same time, the irregularity in the dispersion of carbon atoms within the workpiece will also cause differences in the stacking height on either side of the slot. This asymmetry appears more pronounced as the angle increases. This is so that the abrasive grains gradually cut into the interior of the workpiece as the cutting angle increases. More interstitial carbon atoms are involved in the chip process affecting the dislocation reaction, resulting in an asymmetry in the pile-up height on the workpiece surface. The groove should be regular when the cutting angle is 0, but as the cutting goes on, more atoms build up on the workpiece’s surface, creating a small obscuration above the groove. The radius of the abrasive grain used to cut the atoms on the workpiece’s surface diminishes as the cutting angle rises, resulting in a gradual sharpening and irregularity at the front of the flute.

A comparison of Figure 7 and Figure 8 shows that the existence of a fluid medium influences the distribution and symmetry of the surface build-up height. The fluid medium alters the workpiece temperature and friction coefficient influencing the dislocation response within the workpiece during machining. The defect formation and motion causes the complex mechanisms of plastic and elastic deformation, which is reflected in the surface build-up pattern [33]. As can be seen from the color distribution on Figure 7 and Figure 8, the stacking height of the surface atoms gradually decreases with the increasing cutting angle, while the area where displacement changes occur becomes larger. On the one hand, the reduction in stack height is detrimental to chip removal and, on the other hand, the expansion of the workpiece surface deformation area reduces the surface quality of the workpiece. As the cutting angle increases, so does the depth of cut, resulting in more material damage.

**Figure 10:**
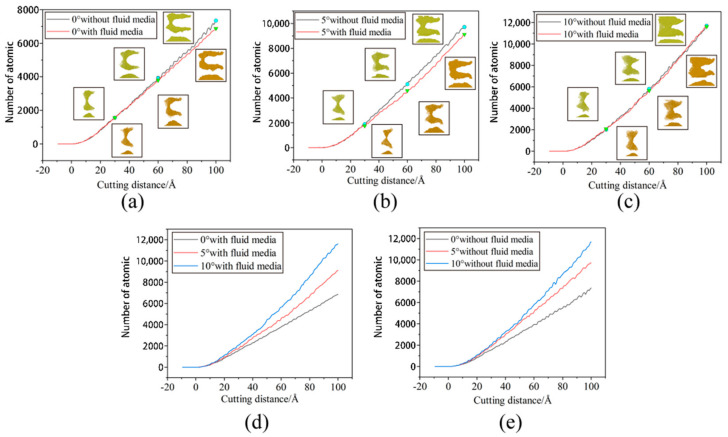
Number of atoms exceeding the workpiece surface. (**a**) θ = 0°; (**b**) θ = 5°; (**c**) θ = 10°; (**d**) wet cutting; and (**e**) dry cutting.

**Figure 11:**
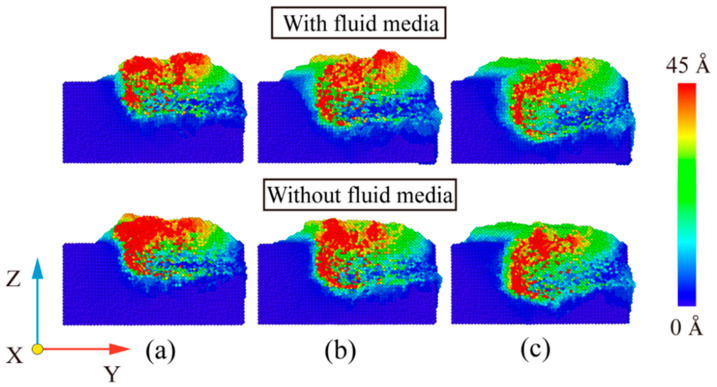
Atomic displacement of cross-section when cutting distance of 10 nm. (**a**) θ = 0°; (**b**) θ = 5°; and (**c**) θ = 10°.

In order to reflect more visually the influence of the fluid medium and the cutting angle on the amount of material removed, the number of atoms beyond the surface of the workpiece during the cutting process was counted, as shown in Figure 10. As can be seen from Figure 9a–c, the fluid medium has a minor effect on the amount of material removed during the cutting process. At cutting angles of 0° and 5°, slightly more atoms are displaced to the workpiece surface during dry cutting. At a cutting angle of 10°, the number of atoms on the surface is nearly the same in both cases. This is explained by the displacement of atoms within the workpiece at a cutting distance of 10 nm, as shown in Figure 11. In the cross-sectional view of the internal atomic displacement of the workpiece when the cutting distance is 10 nm, it can be seen that the atoms are moved into the pile-up on the workpiece surface when the cutting process with a small cutting angle. A large number of atoms with larger displacements do not reach the top at a cutting angle of 10°, but they remain inside the workpiece, and then the upper atoms are squeezed to form pile-up on the workpiece surface. The fluid medium absorbs heat and reduces the thermal movement of the workpiece atoms at small cutting angles, resulting in a slight difference in the number of atoms built up on the surface. However, because the abrasive particle penetrates deep into the workpiece and the atoms in the cutting area are squeezed and moved to the workpiece surface during the large-angle cutting process, they are not very sensitive to the effect of temperature. As shown in Figure 10d,e, the cutting angle has a greater influence on the amount of atomic build-up on the surface. With or without a fluid medium, more atoms within the workpiece are displaced to the workpiece surface as the cutting angle increases. The increase of the cutting angle reduces the surface quality of the workpiece, but it has a significant effect on the material removal to another extent. Figure 11a–c depict the top and side views of the build-up above the workpiece surface at the cutting moment as illustrated in Figure 7 and Figure 8. The surface is free of significant chip deposits during the initial stage of cutting. The surface accumulation of atoms increases in an approximately linear trend as the cutting progresses. The small cutting angle and the presence of the fluid medium reduce workpiece surface damage during the cutting process, which is beneficial for machining.

### 3.3. Analysis of Dislocation Evolution

The dislocations in the iron–carbon alloy workpiece will change with the plastic deformation of the material, and the existence of the dislocations will have a very important impact on the properties of the material. The dislocations in the iron–carbon alloy workpiece will change with the plastic deformation of the material, and the existence of the dislocations will have a very important impact on the properties of the material. In order to investigate the changes of dislocations inside the material during particle microcutting at different cutting angles, the dislocation extraction algorithm (DXA) was used to extract the dislocations inside the workpiece under different conditions of cutting, and the dislocation distribution was obtained for each state. This study found that there is the two dislocations for the Böhler vector b = 1/2 <1 1 1> and b = <1 0 0>, which are in contrast to other dislocations produced by the tool cutting process. The absence of Böhler vectors of 1/3 <1 1 1>, 1/6 <1 1 1> and 1/12 <1 1 1> during the cutting process indicates the absence of a twinning structure. This is consistent with the findings of Katzarov Ivaylo Hristov et al. [34].

**Figure 12:**
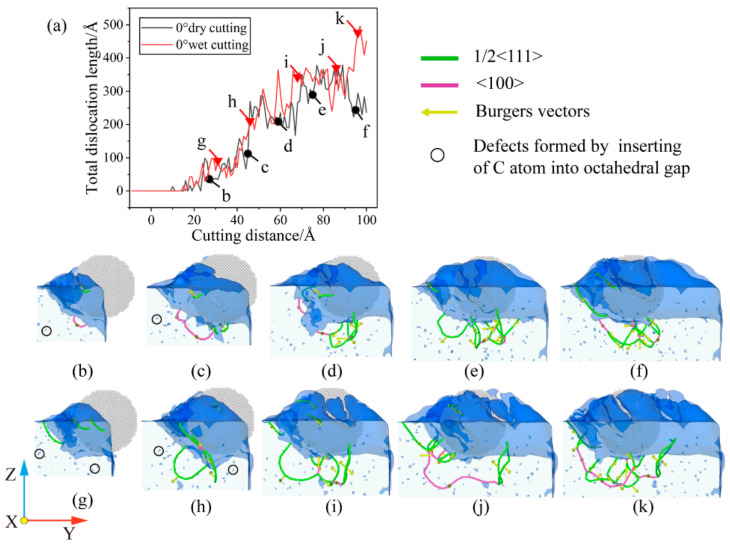
Dislocation evolution under θ = 0°. (**a**) Variation of total length of dislocation with cutting distance; (**b**) cutting distance of 26 Å under dry cutting; (**c**) cutting distance of 45 Å under dry cutting; (**d**) cutting distance of 59 Å under dry cutting; (**e**) cutting distance of 75 Å under dry cutting; (**f**) cutting distance of 95 Å under dry cutting; (**g**) cutting distance of 30 Å under wet cutting; (**h**) cutting distance of 46 Å under wet cutting; (**i**) cutting distance of 69 Å under wet cutting; (**j**) cutting distance of 85 Å under wet cutting; and (**k**) cutting distance of 96 Å under wet cutting.

**Figure 13:**
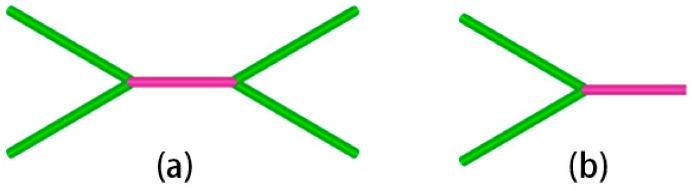
Dislocation structure. (**a**) Double-branch structure; and (**b**) single-branch structure.

When the cutting angle is 0°, the fluid medium does not have a significant effect on the change in the total length of the dislocation, but the dislocation reaction during the cutting process is different. According to Figure 12a, the contact between the abrasive particle and the workpiece in the early cutting stage is an elastic deformation behavior, and there is no dislocation behavior inside the workpiece. First, we analyze the cutting process in the absence of a fluid medium. Before the cutting distance reaches 22 Å, there is a short period of appearance and annihilation of dislocations. In these processes, there is insufficient stress to support dislocation nucleation. When the strain energy reaches a certain threshold, dislocation lines appear one after another. As shown in Figure 12b, a dislocation with Burr’s vector b = [1 0 0] appears below the workpiece. As the cutting progresses, this dislocation develops and serves as a link between the dislocation development in front of and below the abrasive grain. When cutting to about 59 Å, the dislocations in the anterior and posterior areas below the abrasive consist of the structure shown in Figure 13a that we call the double-branch structure. This structure is flanked on the left and right by two dislocations with a Burr’s vector of b = 1/2 <1 1 1> and connected in the middle by a dislocation with a Burr’s vector of b = <1 0 0>. The other structure is called a single-branch structure shown in Figure 13b, which is less than the double-branch structure on one side of the two b = 1/2 <1 1 1> dislocations. Following that, a small part of the deformation damage appears on the lower left of the abrasive, which is considered to be a necessary phenomenon to form the machined surface, which also prevents the development of dislocations at this point. As the cutting progresses to 75 Å, as shown in Figure 11e, the original sub-surface damage is smoothed out, leaving a dislocation of b = 1/2 [1 −1 −1]. The dislocation network under the abrasive is composed of multiple double-branch structures that are intertwined. As the abrasive continues to move forward, new deformation damage gradually begins to occur in front and temporarily prevents the dislocation from reacting. As shown in Figure 12f, the dislocation reaction under the abrasive does not change significantly, while the deformation in the lower left region accumulates strain energy for further dislocation development. For the wet cutting process, the reaction of the two dislocations inside the workpiece is different. As shown in Figure 12g–i, the b = 1/2 <1 1 1> dislocation reaction within the workpiece dominates in the early cutting process. From a cutting distance of 46 Å to 69 Å, the dislocation of b = 1/2 [1 −1 −1] at the lower left of the abrasive is enlarged. At the same time, the dislocation under the abrasive develops and consists of multiple double-branch structures. As the cutting progresses, we notice that the b = [1 0 0] dislocation, which is made up of the b = 1/2 [1 −1 1] and b = 1/2 [1 1 −1], has grown significantly. From Figure 12j, b = [1 0 0] dislocation has become the link connecting the front and lower areas of the abrasive. As the abrasive continues to advance, multiple dislocation lines regenerate below the abrasive and interrupt the previous b = [1 0 0] long dislocations. The dislocations are reorganized, and a new round of dislocation multiplication begins to generate a new processing surface.

**Figure 14:**
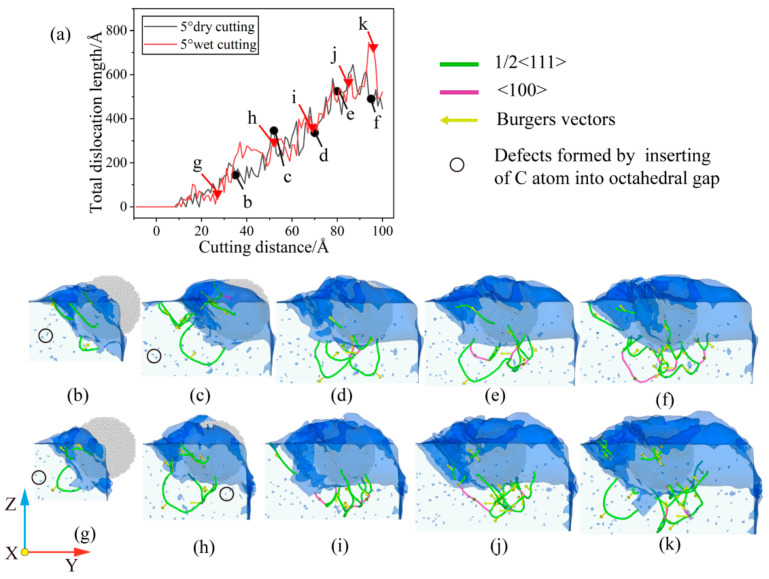
Dislocation evolution under θ = 5°. (**a**) Variation of total length of dislocation with cutting distance; (**b**) cutting distance of 35 Å under dry cutting; (**c**) cutting distance of 52 Å under dry cutting; (**d**) cutting distance of 70 Å under dry cutting; (**e**) cutting distance of 80 Å under dry cutting; (**f**) cutting distance of 94 Å under dry cutting; (**g**) cutting distance of 27 Å under wet cutting; (**h**) cutting distance of 52 Å under wet cutting; (**i**) cutting distance of 69 Å under wet cutting; (**j**) cutting distance of 85 Å under wet cutting; and (**k**) cutting distance of 96 Å under wet cutting.

For cuts with a cutting angle of 5°, the influence of the presence of the fluid medium on the total dislocation length is also not very obvious. As the cutting angle increases, the abrasive grains gradually move into the workpiece. The contact area between the workpiece and the abrasive grains becomes larger, and more atoms are involved in the process, increasing the intensity of dislocation activity on the subsurface. For the dry cutting process, the dislocation activity in the early stage nucleates stably after the cutting distance is 20 Å, and the total dislocation length increases continuously. When the cutting progress reaches 35 Å, several scattered dislocation lines of b = 1/2 <1 1 1> appear around the abrasive grain, as shown in Figure 14b. Among them, the dislocation line of b = 1/2 [1 1 1] in the lower left of the abrasive gradually expands as the cutting progresses. As shown in Figure 14c, when the cutting distance is 52 Å, the dislocation of b = 1/2 [1 1 1] has developed greatly, and the intersection of multiple dislocations on the front and side of the abrasive grain causes dislocation entanglement. As the abrasive advances, the dislocations accumulated in the front transform into a defective sub-surface. The dislocations under the abrasive develop into a small number of single-branch and double-branch structures. Among them, b = 1/2 <1 1 1> dislocations are not only newly generated with the cutting process, but have also evolved from the previous cutting process. As the cutting progresses, the accumulation of strain energy allows the development and reorganization of each dislocation line beneath the abrasive. It can be seen from Figure 14f that the dislocations under the abrasive have been reorganization, and a number of short dislocations with double-branch structures have been newly derived, which prepares the conditions for a new round of growth in the total dislocation length. As can be seen from Figure 14f, the dislocations below the grain have been reorganized, with a dislocation of b = [1 0 0] connecting the dislocation reactions in front of and below the grain. A number of new short dislocation lines with double-branched structures have been derived, preparing the way for a new round of dislocation growth in the total length. For wet cutting, as shown in Figure 14g,h, the dislocation of b = 1/2 [−1 −1 −1] at the lower left of the abrasive is developed. When the cutting distance is 52 Å, the dislocation under the abrasive is a double-branch structure and absorbs b = 1/2 [−1 −1 −1] dislocation. As the cutting progresses to 69 Å, the dislocations under the abrasive gradually proliferate, and a small amount of deformation damage occurs in front of the grain, which prevents the dislocation from extending. As the abrasive advances, as shown in Figure 14j, the small damage and deformation are gradually engulfed by the new deformed layer, and the dislocations under the abrasive grow to varying degrees. At the same time, a long dislocation of b = [1 0 0] connects the dislocation below and the front sub-surface defect. As the cutting progresses, as shown in Figure 14k, deformation and damage reappear on the lower left of the abrasive grain. A dislocation of b = 1/2 [−1 −1 −1] regrows on the left side of the damage, indicating that a new dislocation growth point will appear.

**Figure 15:**
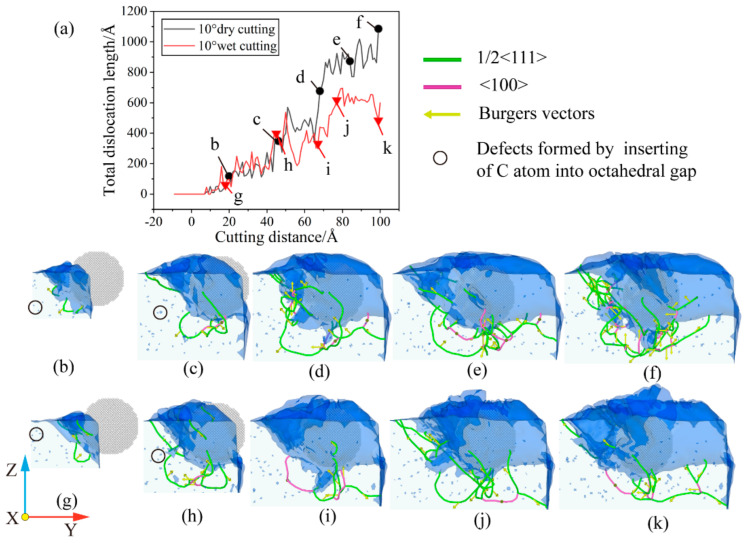
Dislocation evolution under θ = 10°. (**a**) Variation of total length of dislocation with cutting distance; (**b**) cutting distance of 20 Å under dry cutting; (**c**) cutting distance of 46 Å under dry cutting; (**d**) cutting distance of 68 Å under dry cutting; (**e**) cutting distance of 86 Å under dry cutting; (**f**) cutting distance of 100 Å under dry cutting; (**g**) cutting distance of 16 Å under wet cutting; (**h**) cutting distance of 45 Å under wet cutting; (**i**) cutting distance of 66 Å under wet cutting; (**j**) cutting distance of 77 Å under wet cutting; and (**k**) cutting distance of 99 Å under wet cutting.

For cuts with a cutting angle of 10°, the presence of a fluid medium has a greater influence on the total dislocation length in the middle and later stages of the cut. As can be seen in Figure 15a, the total length of dislocations is relatively short in the presence of fluid media. Due to the large cutting angle, the energy exchange between the workpiece and the fluid media is more extensive in the middle and late stages of the cut as the abrasive grains and fluid media penetrate deeper into the workpiece. The fluid medium absorbs some of the energy used to promote the formation of dislocations, resulting in better machining quality and fewer sub-surface defects. For the dry cutting, the total dislocation length steadily increases after the cutting distance is 15 Å. When the cutting distance is 20 Å, a dislocation of b = 1/2 [−1 −1 −1] appears at the lower left of the abrasive, and it develops steadily in the subsequent cutting process. As shown in Figure 15c, the dislocation under the abrasive grain consists of a single and a double-branched structure. The b = 1/2 [−1 −1 −1] dislocation is a component of the double-branch structure and gradually grows. Subsequently, the damage of the abrasive to the workpiece deepens, as shown in Figure 15d, and more short dislocations are derived from the front of the abrasive grains. The original dislocation line under the abrasive has been greatly increased, especially the b = 1/2 [−1 −1 −1] dislocation plays the role of connecting the front and lower dislocations of the abrasive. At this point, the sub-surface of the abrasive’s lower left area has more damage, and the dislocations are derived to a greater extent, so the total dislocation length is rapidly increasing. As the abrasive cuts through the damaged area, the damaged part is smoothed out, and the total dislocation length gradually becomes stable. It is important to note that there are still dislocation lines remaining on the machined surface, which affects the quality of the machined surface. However, when the cutting distance reaches 100 Å, as shown in Figure 15f, new damage appears in the lower left area of the abrasive, which makes the dislocations continue to interweave and multiply. It leads to the formation of a complex dislocation network and heralds the start of a new period of rapid growth. When compared to dry cutting, the dislocation reaction of the cutting process with a fluid medium is less intense. When the cutting distance is 16 Å, a dislocation of b = 1/2 [1 1 1] appears steadily at the lower left of the abrasive grain. As the cut reaches 45 Å, the dislocation below the grain consists of a double-branched structure and absorbs the growing b = 1/2 [1 1 1] dislocation. As the cutting progresses, the damage and deformation layers gradually appear in front and below the abrasive, which also means that the dislocations are reorganized and newly derived. As shown in Figure 15i, damage and deformation in a larger area appear below the abrasive grain, which affects the distribution of the dislocation networks. At this point, there is a single branched structure below the left side of the grain, with a dislocation of b = [−1 0 0] connecting the area in front of and below the grain. As the cut progresses to 77 Å, the defective structure below the grain is smoothed out, while the new damage deformation in front of the grain is deformed by the presence of multiple dislocations. There is a double-branch structure under the abrasive to connect the area under the machined surface and the area under the abrasive. The dislocation of 1/2 [1 −1 −1] is maintained stably at the lower left of the abrasive grain, and the total dislocation length is maintained in a stable state. As the cut reaches 99 Å, the deformed structure beneath the grain is smoothed out and some of the dislocations are obliterated resulting in a decrease in the total dislocation length. This is consistent with the findings of Li Shang-Jie et al. [35]. However, as the cutting continues, the abrasive grains will further damage the workpiece, which will correspondingly initiate the next round of dislocation multiplication.

In order to more intuitively see the changes of dislocation under different cutting conditions, we give the total dislocation length changes of wet and dry cutting under different cutting angles as shown in Figure 15.

**Figure 16:**
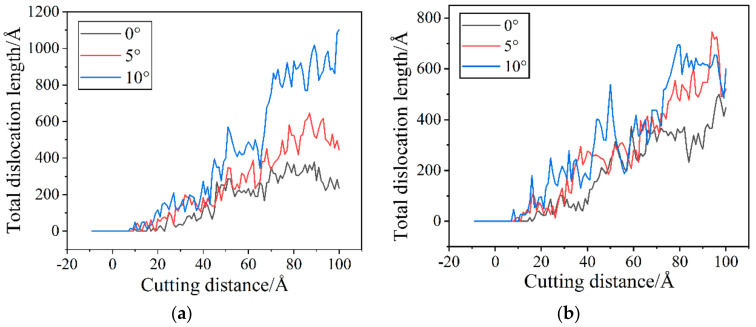
Total dislocation length changes under different cutting angles. (**a**) Dry cutting; and (**b**) wet cutting.

Figure 16a illustrates how the total dislocation length gradually increases as the cutting angle rises. This is because the contact area between the workpiece and the abrasive becomes larger when the cutting angle is large, and more workpiece atoms participate in the machining process. The workpiece temperature rises and dislocation activity becomes more frequent under the influence of energy and stress, ultimately resulting in a large number of defective structures in the sub-surface region of the workpiece. Although the cutting angle has the same effect in cutting processes with a fluid medium, as shown in Figure 16b, the presence of the fluid medium causes higher fluctuations in the dislocation variation at some stages. During dry cutting, as shown in Figure 16a, dislocations become easier to nucleate as the cutting angle increases in the early stage of cutting. This is due to the increased strain energy that the additional atoms engaged in the cut release, which ensures the stress necessary for nucleation. As shown in Figure 16b, dislocations nucleate more quickly in the early stages of cutting for cutting with fluid media. From the temperature distribution curve in Figure 4, it can be known that the early temperature of the cutting process with a fluid medium is relatively high, which easily promotes the nucleation of dislocations. On the other hand, from Figure 12f,k, Figure 14f,k and Figure 15f,k, it can be seen that the cutting process with a large cutting angle has a small amount of dislocation residue under the shaped surface of the workpiece at the end of the cut, which has a certain impact on the quality of the workpiece. According to the analysis above, a smaller cutting angle and the existence of a fluid medium can decrease the quantity of dislocations and the overall length of dislocations, which reduces the generation of sub-surface defect structures.

## 4. Conclusions

In order to reveal the influence of fluid medium and cutting angle on the nano-cutting process, this study establishes a MD model of nano-cutting iron–carbon alloy system with $C_{12}H_{26}$ molecules as the fluid medium. By comparing the dry cutting and wet cutting, the following conclusions are drawn:

1. In comparison to machining without a fluid medium, machining using a fluid medium ($C_{12}H_{26}$) lowers the machining temperature and the coefficient of friction.
2. Temperature and coefficient of friction increase with increasing cutting angle during abrasive flow machining.
3. The cutting angle has a greater influence on the formation of the workpiece’s surface profile and the manner in which the workpiece atoms are displaced, whereas the fluid medium has a lesser influence. When the cutting angle is 0°, 5° and 10°, respectively, the workpiece’s surface profile flows at 45° to both sides. The height of the atomic accumulation on the workpiece’s surface gradually decreases, but at the same time the area where displacement changes occur becomes larger. As the cutting angle increases, so does the depth of cut, resulting in more material damage.
4. The area of displacement gradually expands towards the interior of the workpiece as the cutting angle increases. The number of atoms displaced to the workpiece’s surface decreases and remains within the workpiece. The atoms that accumulate inside the workpiece squeeze the uncut area, causing a bulge in the workpiece’s surface, which degrades the workpiece’s quality, but is beneficial for the removal of large burrs.
5. During the cutting process, a large number of dislocations were discovered at b = 1/2 <1 1 1> and b = <1 0 0>. The b = 1/2 <1 1 1> dislocations dominate, with b = <1 0 0> connecting the dislocations in different areas. The dislocation reaction network is formed by the presence of a large number of single and double-branched structures within the workpiece. During large-angle cutting, the fluid medium can reduce the number of dislocations and the total dislocation length, which in turn reduces the generation of sub-surface defect structures, resulting in better machining quality.

## Figures and Tables

**Table 1:** Model parameters.

Parameters	Values
Workpiece	Iron–carbon alloy
Lattice structure	BCC
Workpiece orientation	[100], [010], [001]
Workpiece size	114.52 Å × 171.78 Å × 85.89 Å
Abrasive particle	SiC
Radius of abrasive particle	25 Å
Atomic number of workpiece	147,773
Atomic number of abrasive particle	6287
Molecular number of fluid medium	62,064

## Data Availability

Not acceptable.
